# MicroRNAs as Critical Biomarkers of Major Depressive Disorder: A Comprehensive Perspective

**DOI:** 10.3390/biomedicines9111659

**Published:** 2021-11-10

**Authors:** Miguel A. Ortega, Miguel Angel Alvarez-Mon, Cielo García-Montero, Oscar Fraile-Martinez, Guillermo Lahera, Jorge Monserrat, Luis Muñoz-Merida, Fernando Mora, Roberto Rodríguez-Jiménez, Sonia Fernandez-Rojo, Javier Quintero, Melchor Álvarez-Mon

**Affiliations:** 1 Department of Medicine and Medical Specialities, University of Alcala, 28801 Alcalá de Henares, Spain; miguel.angel.ortega92@gmail.com (M.A.O.); cielo.gmontero@gmail.com (C.G.-M.); oscarfra.7@hotmail.com (O.F.-M.); guillermo.lahera@gmail.com (G.L.); jorge.monserrat@uah.es (J.M.); luis.munoz@edu.uah.es (L.M.-M.); mademons@gmail.com (M.Á.-M.); 2 Ramón y Cajal Institute of Sanitary Research (IRYCIS), 28034 Madrid, Spain; 3 Cancer Registry and Pathology Department, Hospital Universitario Principe de Asturias, 28806 Alcalá de Henares, Spain; fernando.mora@salud.madrid.org (F.M.); sfernandezr@salud.madrid.org (S.F.-R.); fjquinterog@salud.madrid.org (J.Q.); 4 Department of Psychiatry and Mental Health, Hospital Universitario Infanta Leonor, 28031 Madrid, Spain; 5 Psychiatry Service, Center for Biomedical Research in the Mental Health Network, University Hospital Príncipe de Asturias, 28806 Alcalá de Henares, Spain; 6 Department of Legal Medicine and Psychiatry, Complutense University, 28040 Madrid, Spain; rodriguez.jimenez.psiquiatra@gmail.com; 7 Institute for Health Research Hospital 12 de Octubre (imas 12), CIBERSAM, 28041 Madrid, Spain; 8 Immune System Diseases-Rheumatology, Oncology Service an Internal Medicine, University Hospital Príncipe de Asturias, (CIBEREHD), 28806 Alcalá de Henares, Spain

**Keywords:** Major Depressive Disorder, microRNAs, biomarkers, inflammation

## Abstract

Major Depressive Disorder (MDD) represents a major global health concern, a body-mind malady of rising prevalence worldwide nowadays. The complex network of mechanisms involved in MDD pathophysiology is subjected to epigenetic changes modulated by microRNAs (miRNAs). Serum free or vesicles loaded miRNAs have starred numerous publications, denoting a key role in cell-cell communication, systematically and in brain structure and neuronal morphogenesis, activity and plasticity. Upregulated or downregulated expression of these signaling molecules may imply the impairment of genes implicated in pathways of MDD etiopathogenesis (neuroinflammation, brain-derived neurotrophic factor (BDNF), neurotransmitters, hypothalamic-pituitary-adrenal (HPA) axis, oxidative stress, circadian rhythms...). In addition, these miRNAs could serve as potential biomarkers with diagnostic, prognostic and predictive value, allowing to classify severity of the disease or to make decisions in clinical management. They have been considered as promising therapy targets as well and may interfere with available antidepressant treatments. As epigenetic malleable regulators, we also conclude emphasizing lifestyle interventions with physical activity, mindfulness and diet, opening the door to new clinical management considerations.

## 1. Introduction

Major Depressive Disorder (MDD) is a common and debilitating disease which is characterized by profound physiological, biological and psychosocial changes with detrimental consequences for the affected individuals. In 2008, the World Health Organization (WHO) ranked MDD as the third cause of disease burden, projecting that by 2030 it will be identified as the leading cause [1]. Currently, epidemiological data suggests that the lifetime prevalence of MDD varies from 2 to 21%, being correlated with some sociodemographic factors (i.e., female gender or separated/divorced marital status), child abuse, intimate partner violence, and the presence of physical or mental comorbidities [2]. Notwithstanding MDD is relatively common worldwide and the age of onset is found widely distributed, the prevalence of MDD is superior in high income versus low-middle income countries, generally affecting people in the early adulthood and coursing as chronic-recurrent condition [3]. From an economical perspective, the estimated burden of MDD in 2010 was estimated as $210.5 billion only in the United States [4] with a calculated loss of $36.6 billion per year due decreased workplace productivity and absenteeism resulting in lowered income or unemployment [5]. Furthermore, the real costs of this condition for the affected individuals are virtually extended to the different aspects of their lives, and despite the numerous advances in the knowledge of this disorder, many patients do not improve after the diagnosis, even when treated [6]. More worryingly, there is a link between MDD an increased risk of suicide, where numerous and diverse factors are involved [7].

Most of these risk factors are associated with chronic or prolonged stress situations which induce progressive and detrimental changes in multiple brain structures such as the hippocampus [8]. This chronic stress is related and accompanied with an abnormal immune status, which in turn is associated to an altered interplay with host microorganisms, leading to a sustained neuroinflammation and brain modifications [9,10,11]. Despite the unequivocal role of these factors in the pathogenesis of MDD, today there is no unique and established hypothesis explaining the onset and development of this condition and multiple mechanisms have been described in this sense, therefore confirming the complex nature of MDD. Medical therapies are mainly based on psychotherapeutic interventions as well as on the use of antidepressants, either alone or in combination, although there are other interventions available if needed (electroconvulsive therapy, the use of multiple antidepressants, repetitive transcranial magnetic stimulation, etc.) [12]. In addition, a wide variety of non-medical complementary approaches are being explored in the clinical management of MDD, showing promising results including the use of nutraceuticals [13], mindfulness-based interventions [14] physical activity [15] and sleep interventions [16]. However, about 1 in 3 patients are considered therapy-resistant patients, and it is difficult to find at proper clinical management of these individuals [17]. In addition, there are several difficulties regarding the diagnosis and classification of MDD, as nowadays this is achieved by the assessment of a psychiatric interview and symptoms referred by the patient [18]. Furthermore, the study of multiple clinical markers in MDD are reporting a substantial growing in the last years, in order to identify the therapeutic success of the treatment regimen, also aiding to predict long-term outcomes [19]. The study of micro RNAs (miRNAs) represent a central point in the growing field of epigenetics, being considered as a central point to understand health and disease conditions [20,21]. Thus, the aim of this review is to collect the updated knowledge of the miRNAs more strongly supported by the scientific evidence in MDD, exploring the different links between miRNAs with the onset and progression of MDD, as well as examining promising translational implications in this area.

## 2. A General Overview of Micro RNAs

miRNAs are small endogenous non-coding molecules from 19 to 25 nucleotides, being responsible for gene silencing at post-transcriptional levels [22]. A single miRNA could target hundreds of messenger RNAs (mRNAs), interacting with their 3′ untranslated region (3′ UTR) and repressing protein translation while accelerating mRNA degradation [23,24]. In general, miRNAs are produced in the nucleus, although there is a specific subset of miRNAs which can be synthesized in the mitochondria, known as mitochondrial miRNAs (MitomiRs), although their molecular basis are less established [25]. In the nucleus, there are two principal classifications of miRNA biogenesis designed as canonical and noncanonical pathway. The former start with the transcription of a primary miRNA (pri-miRNA) in a process mediated by RNA polymerases II or III [26,27]. Then, the pri-miRNA is processed into a precursor miRNA (pre-miRNA) by a microprocessor complex, integrated by a ribonuclease III enzyme named Drosha and a double-stranded-RNA-binding protein, known as DiGeorge Syndrome Critical Region 8 (DGCR8) [28]. Subsequently, pre-miRNA is transported from the nucleus to the cytosol through the exportin 5 (XPO5)/RanGTP complex and then is further processed by the RNase III endonuclease Dicer, resulting in a 22 nucleotides miRNA duplex [29]. Eventually, the processing is concluded with the formation of the ribonucleoprotein complex known as miRISC (miRNA-Induced Silencing Complex). The miRISC is composed by one strand of the miRNA, designed “guide strand” Dicer, TRBP (transactivation response element RNA-binding protein), PACT (protein activator of PKR) and Argonaute (Ago) proteins. The complex binds to the mRNA, activating the above-mentioned mechanisms and repressing gene expression. The other miRNA strand, defined as “passenger strand” is often (but not always) removed [30]. The biogenesis of miRNAs is under precise spatial and temporal regulation and it may be regulated at various levels including miRNA transcription; its processing by Drosha and Dicer, in the Ago loading; as well as by RNA editing, methylation, uridylation, adenylation and RNA decay [31]. Moreover, there are noncanonical pathways in which miRNAs may be synthesized in a Drosha/DGCR8 or Dicer independent manners. The role of some introns (named mirtrons), small nucleolar RNAs (snoRNAs), endogenous short hairpin RNAs (shRNAs) and transfer RNAs (tRNAs) must be here highlighted, although this field is growing rapidly, and these are not the only mechanisms reported [32,33]. Frequently, target mRNAs and miRNAs are located in the cytoplasm, however there is also evidence of miRNA/mRNAs silencing in the rough endoplasmic reticle (RER), the trans Golgi network (TGN), early and late endosomes, lysosomes, stress granules, processing bodies, multivesicular bodies and of course in the proper nucleus and mitochondria [29]. Hence, miRNAs are involved in the regulation of almost every cellular process, being considered as a central mediator of cell phenotype and adaptation to the environment [34].

There are several forms in which miRNAs may be studied. Notwithstanding miRNAs often act in the cells where they are produced, these miRNAs may be found at extracellular levels. At these point, mature miRNAs could appear bound to the Ago protein, or encapsulated either in microvesicles/exosomes form, apoptotic bodies or HDL particles [35]. These miRNAs are nuclease-resistant entities and they have been found in almost each body fluid, including plasma, saliva, urine, cerebrospinal fluid, amniotic fluid, tears, breast milk and semen, among others [36]. Furthermore, extracellular miRNAs are thought to be mostly byproducts of cellular activity, probably involved at some extent in cell-to-cell communication [37]. Thereby, the study of miRNAs could be achieved either at cellular/tissue levels or any corporal fluid. Extraction and purification methods include different commercial kits and products such as phenol: chloroform extraction followed by alcohol precipitation (TRIzol), phenol:chloroform followed by solid-phase extraction (column-based; miRVana and miRNeasy) and solid-phase separation with/without affinity resin (Norgen total and Isolate II). However, there has been reported some differences in these methods and further efforts are needed in order to refine available kits and protocols [38]. Regarding miRNAs expression, the main approaches used in their detection are real-time quantitative PCR (RT-qPCR), in-situ hybridization, microarrays and RNA-sequencing, whereas the available technologies used to recognize mRNAs targets include in silico predictive models, transient transfection with miRNA mimic/miRNA antagonist (Better using lentivirus or plasmid transfection), CRISPR/Cas9 technologies and induced RNA/Protein cross-linking followed by immunoprecipitation, digestion and amplification [22].

In this case, miRNAs nomenclature is perhaps an entangling and complex point in current and past investigations in this field. As novel discoveries in the molecular biology of miRNAs are frequently arising, it is important to follow and use a global nomenclature for designing the miRNAs analyzed. A mature miRNA is designed as MIR-X, being X the gene responsible for the miRNA transcription, although it is frequent to find in the literature miR-X as well. Now, it is widely accepted the differentiation between miR-X-3p and miR-X-5p, as it may be codified from the 3′or 5′arm. pri-miR-X is the primary miRNA and pre-miR-X the precursor miRNA. Moreover, miR-Xy refers to the different members of the miR-X family (y = a letter or a dot-number). Identical miRNAs sequences encoded by distinct genomic loci may be referred as miR-Xy-1/2/3. Finally, there are other entities such as miR-XOS (mirror miRNA gene of the miR-X), LOR-X (loop fragment of the miRNA precursor), MOR-X (microRNA-offset RNAs) and IsomiRs which entails their own classification and nomenclature [39]. Currently there are databases and scientific reviews directed to address this issue, as this will bring quicker progress in this area [40]. In this study we will collect past knowledge of some miRNAs with possible nomenclature limitations, and we encourage for an adequate use of miRNAs designation in future studies.

Due to their implication in multiple cellular and physiological processes, miRNAs have been established as promising biomarkers with suggested applications as diagnostic tools, prognostic or predictive markers as well as critical agents to understand the entire pathophysiology of different psychiatric disorders such as MDD, having been proposed as potential therapeutic targets [41,42,43,44,45]. In this sense throughout this review, we will analyze the implication of miRNAs with special focus on their pathogenesis, diagnosis, prognosis, and therapy of such an intricated disease.

## 3. miRNAs Involved in the Pathogenesis of MDD

The pathophysiology of MDD is quite complex. Traditionally, monoamine hypothesis was considered the most plausible cause of MDD. This theory consists of the altered functioning of the monoamines in the brain: Serotonin (5-HT), dopamine (DA) and norepinephrine (NE), which are considered major therapeutic targets of many antidepressants [46,47]. However, despite the involvement of these components in the pathogenesis of MDD, this explanation is not far enough to address some critical issues such as why antidepressants may have a delayed or partial response and in some cases an absence of response [48]. Then, multiple complementary and plausible hypothesis have been developed in this field. For instance, it has been demonstrated the relevance of other neurotransmitters such as the excitatory glutamate and the inhibitory gamma-aminobutyric acid (GABA) and the neuropeptide substance P [49]. Furthermore, there is growing evidence supporting the implication of a disrupted circadian rhythms and of its master regulator, melatonin in the onset and development of MDD [50]. Other studies have proposed the central role of an altered hypothalamic-pituitary-adrenal (HPA) axis in MDD, placing the stress circuit as one of the major pathophysiological mechanism in this disease [51]. An abnormal response to stress may lead to substantial changes in the different structures of the central nervous system, particularly in cortical brain areas (dorsal and medial prefrontal cortex, the dorsal and ventral anterior cingulate cortex, the orbital frontal cortex and the insula), subcortical limbic brain regions (hippocampus, amygdala and the dorsomedial thalamus), brainstem and basal ganglia, affecting their structure and functionalities and even their connectivity [52]. These regions show an impaired neurogenesis and neuroplasticity [53]. In accordance with this fact a disrupted activity of different neurotrophins such as brain-derived neurotrophic factor (BDNF), is frequently related to MDD and other psychiatric disorders, being responsible for orchestrating many critical neuronal processes (i.e., by promoting neural development, survival, neuroplasticity, and neurogenesis) [54]. Moreover, an exacerbated neuroinflammatory response is crucial to understand the pathophysiology of MDD. In this sense, multiple inflammatory markers may cross the blood brain barrier (BBB), activating the neuroglia and dysregulating multiple inflammatory mediators such as kynurenine pathway, with neurotoxic effects [55]. The boosted inflammatory response might be triggered by an altered status of the gut microbiota (dysbiosis) and an enhanced bacterial translocation in the bloodstream [56,57], although these microbials might also influence in the MDD pathogenesis by other mechanisms via the vagus nerve, endocrine or metabolic factors, constituting the well-known microbiota-gut-brain (MGB) axis [58]. An exacerbated production of Reactive oxygen species (ROS) and reactive nitrogen species (RNS) and a decrease of antioxidants levels lead to an abnormal cellular condition which is known as oxidative stress (OS). It is well-known that OS is a prominent agent involved in the pathogenesis of MDD, as the brain is a region particularly susceptible to the oxidative damage [59,60]. Last but not least, a wide variety of cell signaling pathways are disrupted in the different brain areas of patients with MDD such as PI3K/Akt/mTOR, MAP kinases (MAPK), Wnt/βcatenin and many others [61].

**Figure 1:**
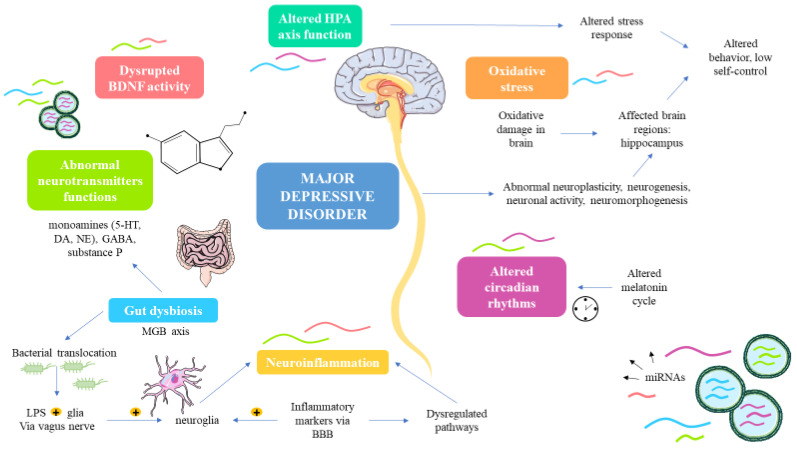
Mechanisms involved in the etiopathogenesis of MDD in which miRNAs may be key mediators BBB: blood brain barrier. 5-HT: serotonin. DA: dopamine. NE: norepinephrine. GABA: gamma-aminobutyric acid. MGB axis: microbiota-gut-brain axis. HPA axis: hypothalamic-pituitary-adrenal axis. Kyn: kynurenine. LPS: lipopolysaccharide.

In this context, miRNAs might be critical regulators of many of the targets and factors implicated in the pathogenesis of MDD, having been related among others to neuroinflammation, altered neurogenesis, neuroplasticity, stress response or circadian rhythms, as proven in different animal models [62]. In Figure 1, we summarize the different mechanisms by which miRNAs may participate in the pathogenesis of MDD. However, for facilitating the reading, we have classified three topics to be described in this context, miRNAs regulating neurotransmitters and neuropeptides, miRNAs involved in stress and brain changes and miRNAs involved in neuroinflammation and MGB axis dysfunction.

### 3.1. miRNAs Involved in Neurotransmitters and Neuropeptide Dysregulation

As above mentioned, aberrant monoamines and neurotransmitter functioning is a central feature of the pathophysiology of the disease. miRNAs are critical mediators of neurotransmitters and neuropeptide systems and their disruption appears to be related to the abnormal performance of these components [63]. In the field of MDD there has been described different miRNAs affecting both monoamine and non-monoamine neurotransmitters. Serotonin is the main target of multiple antidepressants and previous studies have demonstrated the role of miRNAs in the whole serotoninergic system has been widely studied in the brain and other tissues [64]. For instance, miR-15 and miR-16 cluster, located at the human chromosome 13q14.3 has been proven to modulate the expression of serotonin transporters (SERTs) in rat brain raphe RN46A and human placental choriocarcinoma JAR cells [65]. Yang et al. [66] demonstrated that miR-16 is involved in the regulation of apoptosis and autophagy in these rodents and that this miRNA could partially mediate the success of Selective serotonin reuptake inhibitors (SSRIs). Interestingly, there are certain polymorphisms that may drive to an abnormal miR-15/miR-16 functioning. The variant rs28457673, affecting to miR-15/16/195/424/497 family appears to induce an aberrant signaling of insulin-like growth factor receptor 1 (IGF-1R) and MAP kinase (MAPK) pathway, which may be related with the appearance of post-stroke depression (PSD) [67], a poorly understood condition involving approximately one third of stroke survivors [68]. Another miRNA probably related with the altered serotonin function in patients with MDD are miR-30e, miR-34a and miR-135. The former miRNA has been found to be upregulated in the postmortem brain of depressed patients died by suicide, and it seems to be a regulator of Zdhhc21, a major palmitoyl acyltransferase of the serotonin receptor 5-HT1AR [69]. The decreased palmitoylation of 5-HT1AR due to the decreased expression of Zdhhc21 by miR-30e, appears to be an important mechanism involved in the pathophysiology of MDD, and future strategies may be directed to target this network. Iacono et al. [70] showed that under chronic stress exposure, increased levels of miR-34a dysregulated serotonin transmission, targeting different components of the serotoninergic pathway in the raphe dorsal nuclei of mice, enhancing a depressive-like behavior. In the same manner, miR-135 is considered an essential marker of mood, aiding in the regulation of serotoninergic neurotransmission [71]. In patients with MDD, miR-135 is prominently downregulated in the blood and in the raphe nuclei of suicide depressed patients in comparison to healthy controls, what could have negative consequences both at the antidepressant efficacy and the serotoninergic activity [72]. On the other hand, there are also different miRNAs involved in the dysregulation of DA, which is frequently associated with anhedonia (loss of interest or pleasure), a core symptom of MDD [73]. Lethal-7 (Let-7) miRNAs, a group of miRNAs downregulated in MDD has been tested in previous studies due to its regulatory role on Dopamine 3 receptor (D3R). The role of D3R in depression is complex, and either a deficient or excessive functioning of this system appears to be negative in the pathophysiology of MDD. Thus, D3R antagonism/partial agonism in provides an antidepressant effect in stressed rodents [74]. In this sense, Let-7d may interfere with D3R expression in the brain and it could be an interesting approach to prevent the anxiogenic and pro-depressant effects of D3R overexpression [75]. Similarly, miR-504 is a central posttranscriptional regulator of D1R [76] and of D2R in the nucleus accumbens, leading to significant alterations in the functioning of these neurotransmitters [77]. Furthermore, miR-1202 is another dysregulated miRNA in patients with MDD, and its main target is the metabotropic glutamate receptor 4 (GRM4), which modulate not only serotoninergic and dopaminergic but also glutamatergic and GABAergic transmission [78]. Likewise, miR-29b-3p, a downregulated miRNA in MDD also target GRM4, as well as controlling Ca2+ influx. Ketamine, a main antidepressant seems to act partially through the augmentation of this miRNA, favorably contributing to cell survival, cytodendrite growth and raised extracellular glutamate concentration in vitro. [79]. Additional miRNAs targeting glutamate transmission is miR-101b, downregulated in the prefrontal cortex of a genetic animal model of depression, which inhibit the glutamate transporter SLC1A1 (also known as EAAC1 or EAAT3), upregulated in depression [80]. The overexpression of miR-124 observed in patients with MDD also appears to modulate the function of several glutamatergic components, including Gria3 (glutamate receptor, ionotropic, Ampa3), Gria4 (glutamate receptor, ionotropic, Ampa4), Grin2a (Glutamate Receptor, Ionotropic, N-Methyl D-Aspartate 2A), Grin2b (Glutamate Receptor, Ionotropic, N-Methyl D-Aspartate 2B) [81]. Indeed, previous studies have found worrisome association between GRIN2B and GRM2 and suicide, and the female sex is related to higher expression of these, and the rest of the glutamatergic elements controlled by miR-124 [82], although to our knowledge there are no studies comparing miR-124 expression among both sex, which may aid to explain the observed differences. Finally, miR-451, downregulated in the hippocampus of patients with MDD, also appears to participate in the expression of the GABAergic receptor (GABAA receptor associated protein) and cholinergic neurotransmission (muscarinic cholinergic receptor 5) [83].

Regarding neuropeptides, current research has failed to find an association between miRNAs and melatonin dysregulation in patients with MDD. However, there has been recognized some miRNAs implicated in the altered circadian rhythms in depressed individuals, as it is the case of miR-182, a modulator of the CLOCK gene. Saus et al. [84] reported that those individuals carrying the T allele of the rs76481776 polymorphism could lead to an aberrant processing of pre-miR-182, which may contribute to the dysregulation of circadian rhythms in MDD patients with insomnia through the dysregulation of CLOCK and other critical genes. Satyanarayanan et al. [85] found that after prolonged stress exposure, augmented levels of miR-200a were involved with a plethora of negative effects and a disrupted circadian rhythm, probably being implicated in the coexistence of pain and depression. Sirtuin-1 (SIRT-1) is a crucial enzyme downregulated in MDD, being associated with OS, decreased neurogenesis, neuroglia dysfunction and more prominently with circadian disruption [86,87,88]. Among the altered miRNAs described in MDD targeting SIRT-1 it is of note the role of miR-124 [89], miR-134 [90], miR-138 [91] and miR-155 [92], offering potential targets to increase the expression of SIRT-1 in MDD. Eventually, miR-24 upregulation, observed in patients with MDD downregulates the expression in the hypothalamus of oxytocin, a pivotal neuropeptide linked to the onset and development of MDD [93,94]. Overall, there are multiple miRNAs involved in the abnormal functioning of neurotransmitters and neuropeptides in patients with MDD, although they may take part in other mechanisms that will be subsequently discussed.

### 3.2. miRNAs Related to Stress and Structural, Functional and Molecular Changes in the Brain

There is a growing amount of evidence placing stress as a decisive factor of MDD onset and progression. Indeed, it is now accepted that there are certain polymorphisms responsible for an increased vulnerability of suffering from depression, and together with early life stress (ELS) and prolonged (chronic) stress may drive to a particular phenotype in the central nervous system. This consist of an increased HPA axis activity, including an elevation of the stress hormone, cortisol inducing a molecular susceptibility. Simultaneously, there is a cognitive and emotional vulnerability derived from all these factors that eventually may lead to the appearance of depression [95]. In this context, miRNAs are relevant mediators of cellular response to the stress conditions, leading to a restore or reprogramming of their genes either by modulating the expression of certain miRNAs, or the activity/mode of action of miRNA-protein complexes [96]. This means that miRNAs are part of the adaptative response of the cell to the stress and they may play a major role in the development of MDD specially after early-life and chronic stress exposure. Thus, Allen and Dwivedi [97] examined the potential miRNAs related to ELS and their relationship with MDD and suicidal behavior. They found that despite little studies have been conducted in this field, miR-16, 29, 124, 125 and 200 were the most frequent miRNAs altered after ELS either in the brain or blood samples. In vivo models have demonstrated the role of these miR-16 in the brain response to stress. On the other hand, miR-16 overexpression in the hippocampus appears to have a negative impact in depressive rats induced by maternal deprivation (MD), a model of ELS, leading to decreased expression of BDNF [98]. Similar results were obtained in the prefrontal cortex of rats exposed to inescapable stress during their adolescence [99]. However, these results were not extrapolated to chronic unpredictable mild stress (CUMS) rodents, which are the most used, effective and reliable animal model [100]. This may indicate a differential regulation of miRNAs between early and chronic stress induced depression, which may be considered in the etiopathogenesis of MDD. On the other hand, miR-29 is largely expressed by astrocytes whereas miR-124 is highly expressed in neurons [55], suggesting that ELS may impact to the normal regulation of the different cell populations in the brain. The former, miR-29 is intimately related to the endoplasmic reticulum stress, which is clearly implicated in the pathophysiology of MDD [101,102]. The latter, miR-124 is a promising target of glucocorticoid receptor (GR), also modulating a plethora of cell and molecular targets involved in stress response and neuroplasticity [103,104]. The role of miR-125 in MDD is more elusive, and it seems to augment in acute stress exposure and decrease in CUMS models, also participating in the neural plasticity in a negative fashion [105]. MiR-200 comprise a group of miRNAs (miR-200a/b/c) that appears to be upregulated in learned helplessness (LH) in comparison to non-learned helplessness (NLH) rats, an animal model of stress-induced behavioral depression [106] Importantly, not only mir-200 but also other miRNAS such as miR-96, miR-141, miR-182, miR-183 and miR-198 were shown to be overexpressed in LH rats, all belonging to a shared polycistronic loci, hence suggesting that the coordinated regulation of these miRNAs are at transcriptional levels. Conversely, there are other miRNAs implicated in stress resilience. Furthermore, the upregulation of miR-504 and the downregulation of D2R in the nucleus accumbens of MD/CUMS rats enhanced enhances behavioral vulnerability to stress during adulthood [77].

Regarding chronic stress it is of note that this condition is related not only to the development of MDD but also with substantial changes in the structural, functional, synaptic and cellular/molecular neuroplasticity [107]. Moreover, chronic stress negatively alters the process of neurogenesis, which is prominently conducted in the hippocampus [108]. Conversely, an increased neurogenesis may be related to stress resilience and improved response to stressful conditions, which may be modulated either by intrinsic factors (BDNF, neurotrophic factors, inflammatory cytokines, transcriptional programs, neurotransmitter and hormones) or extrinsic (physical activity, diet and stem cell therapy) [109]. miRNAs are major mediators of these factors, also exerting important implications in neuroplasticity and stress related responses [110]. Volk et al. [111] claimed the importance of amygdalar miR-15a in stress adaptation and a reduced expression of this miRNA could play a detrimental role in stress-related psychopathologies. They showed that after exposure to chronic stress, this miRNA was increased, targeting the FK506-binding protein 51 (FKBP51), which is a critical driver of MDD and a potential therapeutic target [112]. On the other hand, reduced levels of miR-15a in the amygdala are related to anxiety and abnormal responses to stress [111]. Other studies have denoted the relevant role of miR-30 family in the phenotyping of chronic stress-induced depression, targeting epigenetic and transcription regulators (M113 and Runx1) as well as cell signaling mediators (Socs3, Ppp3r1, Gpr125 and Nrp1), thereby altering hippocampal neurogenesis and neuroplasticity [113]. Similarly, miR-134 has also been proven to be upregulated in the ventromedial prefrontal cortex of CUMS rats, downregulating Limk1 and cofilin, leading to an abnormal synaptic/structural plasticity and a depressive-like phenotype [114]. Furthermore, Zhou et al. [115] showed miR-183, miR-202, miR-493 and miR-3573 were upregulated in the hippocampus of CUMS rats whereas miR-370 was prominently downregulated. Recently, Torres-Berrío et al. [116] reported that reduced levels of miR-218 in the medial prefrontal cortex enhanced susceptibility to chronic stress. Interestingly, they showed that miR-218 levels in blood corresponded with those observed in the hippocampus either when upregulated or downregulated. Yoshino et al. [117] noticed that there was a unique signature of miRNAs and an upregulation of their main processing enzymes (DROSHA, Dicer) in the dorsolateral prefrontal cortex (dlPFC) of depressed patients. They found a shift in the expression of miR-376c-3p, miR-455-3p and miR-337-3p in the synaptic fraction over total fraction in MDD subjects compared with healthy controls. These miRNAS were mainly related to synaptic plasticity, nervous system development, and neurogenesis, therefore elucidating the vital role of miRNAs in the neuroplasticity of depressed patients. On the other hand, animals resilient to stress seem to downregulate miR-18a-5p and increase HT1AR expression whereas animals behaviorally vulnerable to stress did not show this mechanism [118]. Moreover, augmented levels of miR-18a together with increased miR-124a expressionn are implicated in the downregulation of the GRs, which appears to be relevant to depressive-like behaviors [119]. In summary, there are multiple ways in which miRNAs cam participate in the response and effects of stress in the organism, initiating or aggravating the development of MDD.

Another major determinant of the neural changes observed in the CNS of patients with MDD relies on BDNF, and many miRNAs have been recognized as important regulators of this and other neurotrophins. For instance, as previously reviewed, one of the main targets of miR-16 is BDNF, responsible for the induction of depressive-like behaviors induced by ELS [98]. Zhao et al. [120] reported that the ventrolateral orbital cortex of CUMS rats showed an increased expression of dual-specificity phosphatase 1 (DUSP1) and decreased ERK and BDNF expression. Importantly, the overexpression of DUSP1 was decreased miR-101 expression. Conversely, augmented levels of miR-101 were able to inhibit DUSP-1 activity, hence augmenting the expression of ERK and BDNF. In the same manner, miR-144 is another crucial miRNA downregulated in the hippocampus CUMS rats [121]. Its main target associated to its possible neuroprotective role is PTP1B, responsible for inhibiting TrkB/BDNF signaling. Additionally, miR-133b is another miRNA with promising antidepressant effects. Despite being poorly expressed in the hippocampus of depression rat models, if overexpressed it may decrease the expression of the connective tissue growth factor (CTGF), preventing cell apoptosis of hippocampal neurons, and neuroinflammation while increasing the expression of GFAP, BDNF and other neurotransmitters [122]. At the same time, there are other miRNAs overexpressed responsible for BDNF downregulation as it is the case of miR-124 is upregulated in the hippocampus of CUMS rats, while BDNF and CREB-1 are downregulated, what may be reversed with the inhibition of miR-124 [123]. In a similar way, miR-221 is also responsible for the downregulation of BDNF and CREB-1, acting directly through Wnt2 [124]. Moreover, it was observed that depressed patients with type 2 diabetes mellitus display augmented miR-128 and cortisol blood levels associated to reduced levels of BDNF and shortened telomeres [125]. Thus, the synergic action of different miRNAs and stress may influence an accelerated aging in those patients, which may be mediated by a decrease in different trophic factors such as BDNF. Furthermore, higher serum miR-132 and miR-182 were also related to decrease BDNF levels [126]. Not only BDNF is a target of different miRNAs but also ciliary neurotrophic factor (CNTF) appears to be regulated by miR-155 [127]. CNTF is another critical mediator of the hippocampus activity and function and an altered functioning of this component is related to anxiety and depressive behaviors [128].

Finally, an altered cell signaling is a central characteristic of many diseases and previous studies have claimed the relevance of miRNAs in the hyperactivation or suppression of different molecular pathways [129,130,131]. In the field of MDD, Ferrúa et al. [132] described a set of putative targets of various miRNAs overexpressed and downregulated, showing that 54 miRNAs and up to 29 union pathways were statistically significant in patients with MDD in comparison to non-depressed individuals. These targets included PI3K/Akt pathway (Let7b/c miR-26a-3p or miR-200) [85,133,134] whose dysregulation in MDD is related to a deficient neurotransmission, neuroprotection and neuroinflammation [135], MAPK (miR-15, miR-16, miR-24-3p, miR-146a/b and miR-425) [67,136], Wnt (miR-128-3p, miR-155 and MIR-221) [124,137,138] and other less recognized areas related to focal adhesion, TGF-β, ErbB or prolactin signaling pathways, pluripotency of stem cells, proteostasis, steroid biosynthesis, viral or bacterial infections, carcinogenesis and neurodegenerative diseases [132].

### 3.3. miRNAs Associated to Neuroinflammation and MGB Axis

Systemic inflammation with increased production of several cytokines and local neuroinflammation are central characters of MDD, opening a plethora of potential therapeutic windows [139,140]. miRNAs are central players of the inflammatory reactions occurred in MDD, mediating with the crosstalk between neurons, neuroglia and immune cells [55]. Sun et al. [141] studied the role of miR-96 overexpression in the CA1 of the hippocampus. They observed that miR-96 inhibited synaptic vesicle glycoprotein 2C SV_2_C, promoting OS and inflammation characterized by increased MDA, TNF-α, and IL-1β release. Sun et al. [142] explored the role of miR-155 in the neuroglia and the hippocampal neurons. Meaningfully, they observed that increased expression of this miRNA was related to a decreased survival of BV-2 microglial cells and reduced viability of this population, leading to a decrease survival of HT22 hippocampal cells in vitro. Among the main targets of miR-155-treated microglial cells they found thar there was a dysregulation of the apoptotic regulators Bax, Bcl-2, pro-caspase-3 and cleaved-caspase-3. In addition, miR-155 also modulates TLR, IL-1R, TNFR, and interpheron-alpha receptor (IFNAR)-mediated signaling pathways, which are critical molecules upstream of the inflammasomes, a central orchestrator of the MDD immunopathogenesis [143,144]. Furthermore, the regulatory role of miR-200a in some crucial signaling pathways related to MDD and inflammation such as PI3K or JAK/STAT routes [85]. On the other side, there are downregulated miRNAs in MDD also playing crucial roles in the regulation of the inflammatory response. This is the case of miR-124 targeting important OS and inflammatory cytokines through its inhibitory actions on the signal transducer and activator of transcription 3 (STAT3), a signaling route related with increased production of nitric oxide synthetase and proinflammatory cytokines, including IL-6, IL-1β, TNF-α, and MCP-1 [145]. As described before, miR-133b through the suppression of CTGF may also diminish the inflammatory damage in animal models of depression, encouraging for the use of this miRNA in future therapeutic approaches [122]. Simultaneously, miR-135a, an important miRNA dysregulated in MDD may also mediate anti-inflammatory actions through targeting IL-1β, IL-6 and TNF-α [146] as well as TLR4, a major hallmark of the neuroinflammatory process that may be related to stress and MDD [147]. Analogous results were obtained with miR-144-5p, whose levels were inversely related to the reduction of 17 inflammatory proteins following antidepressants therapy [148]. Mir-146a is a master regulator of ionized calcium binding adapter molecule 1 (Iba-1), a molecule upregulated in MDD associated with an increased level of inducible nitric oxide, IL-1β, TNF-α, interleukin 1 receptor associated kinase 1 (IRAK1), TNFα receptor-associated factor 6 (TRAF6) and phosphorylated NF-κB p65 [149].

Gut dysbiosis is another potential link between inflammation and MDD. Nowadays, it is undeniable that gut microbiota metabolites shape host immune system and can travel via vagus nerve to modulate behavior and mood [150], nevertheless, little is still known about the interaction with host miRNAs expression. Preclinical animal models with germ-free (GF) mice have demonstrated that gut microbiome strongly affects brain areas related to behavior. What it was certainly unknown was if microbiome could shape host microRNA synthesis. Indeed, some results in GF mice suggest that microbiome can modulate miRNA expression in amygdala and prefrontal cortex [151]. More research groups are starting to support the hypothesis of miRNAs-microbiota networks in psychiatric disorders. In a study, it has been seen that GF vs colonized animals show different miRNAs expression, involving CREB and Ras/MAPK pathways, with relevant signification of miR190a in MGB axis [152]. Other studies corroborated that gut microbiota may have an important impact on the miRNA expression levels, and specially in hippocampus [153]. Moreover, the presence of lipopolysaccharides (LPS), a critical marker of gut dysbiosis in the bloodstream is also responsible for the induction of a depressive phenotype through the activation of the indoleamine 2,3-dioxygenase (IDO) enzyme [154]. Recently, it was demonstrated that miR-874 was involved in the homeostatic response of the brain to LPS. Suento et al. [155] showed that LPS augmented the expression of both IDO and miR-874-3p in the prefrontal cortex of LPS-induced depression-like behavior in mice. They show that this miRNA was mainly directed to inhibit the activity of IDO, reducing the exacerbated activity of this enzyme. Indeed, they found that combined action of this miRNA plus an additional IDO inhibitor may be sufficient to prevent the LPS induced depression.

In a study some pups were assessed showing that early life stressful experiences promote anxiety and lower gut microbiota diversity. In other phase they proved the effects of *Lactobacillus paracasei* supplementation demonstrating helpful effects in stressed rats with proper regulation of neurotransmitters levels (DA, 5-HT, NE), stress hormones (corticosterone) and GRs. The downregulated miR-124 and upregulated miR-132 in stressed animals did not changed in expression with supplementation, but due to their connection with GRs and glutamate receptor GluR1, the probiotic intake was proposed to prevent early life stress [156]. Another research work with C57BL/6 mice fed with Bifidobacterium longum showed different relative expression of miR-652 in brain, and also of its target Dab1, which is a protein involved in brain development and cognitive function. Moreover, this probiotic species had shown improved behavior and sociability in BALB/c mice [157]. Furthermore, there is a promising interventional approach here through diet, as it may critically affect both gut microbiota and immune system crosstalk [158]. Differently, some authors have reviewed that food-derived miRNAs (xenomiRs) have an impact on health. Gut microbiota composition and intestinal permeability status determine the interplay with these xenomiRs, so literature suggest that dietary miRNAs may modulate microbiome functions [159]. However, there is still no evidence about the effects of these exogenous miRNAs in MDD pathophysiology, and due to the multiple effects of miRNAs and Mediterranean diet we encourage for further research in this field.

**Figure 2:**
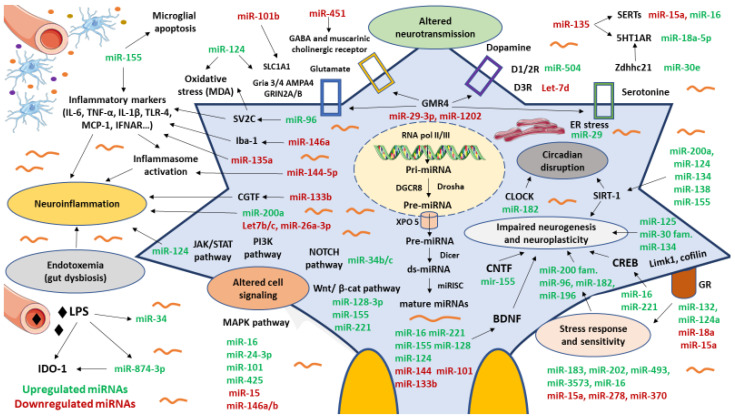
A general picture of the molecular background of miRNAs in MDD. As showed, there are multiple pathways affected by the dysregulation of a set of miRNAs in this mental disorder. Some of their molecular targets are involved in critical pathophysiological mechanisms of MDD, and either the upregulation (represented in green) or downregulation (In red) may be partly responsible for the changes in the neurons and glial cells of depressed individuals. Some of these alterations have been reported in critical areas of the brain such as the amygdala or the hippocampus, clearly implicated in the etiopathogenesis of MDD. Finally, there are some miRNAs that may be regulated by the exposure to the pathological environment including after acute or prolonged stress or due to the endotoxemia and neuroinflammation.

In Figure 2, we show the critical role of miRNAs in the molecular and cellular changes as well as some of their putative targets.

## 4. miRNAs with Diagnostic, Prognostic and Predictive Value

Despite cumulative advances made in the field of MDD there is an urgent need to integrate novel dimensions in the diagnostic criteria, also looking for novel predictors of therapy response and disease progression [160]. As above mentioned MDD diagnosis is based on the symptomatology reported by the patients and assessed through a psychiatric interview. In this sense, the most important clinical practice guideline is the Diagnostic and Statistical Manual of Mentalgluco Disorders 5 (DSM-5). For being diagnosed as MDD, an individual may present at least 5 or more symptoms considered in this manual for ≥2 weeks [161]. Of them, one must be either depressed mood or anhedonia named as main criteria. Secondary symptoms include, among others, appetite or weight changes, fatigue or loss of energy, psychomotor agitation or retardation, sleep difficulties (insomnia or hypersomnia), feeling of excessive guilt or worthlessness, diminished concentration capacities and suicidality [162]. According to these criteria, MDD could be diagnosed as mild, moderate and severe depression, although there is no consensus regarding the specific symptomatology typical of each type. Thus, it is important to consider other systems such as the Hamilton depression rating scale (HAM-D) [163]. Simply, an individual might be classified as non-depressed no depression when pointed from 0 to 7, mild depression (between 8–16); moderate depression (17–23); and severe depression (≥24) [164]. Although this method is considered as the gold standard to measure MDD severity it presents some issues, especially regarding scoring difficulties and psychometric weaknesses [165]. Hence, during the six decades from the first proposal of this classification, multiple studies have been developed by creating improved protocols for this scale, converting this method in general lines as a feasible and objective measure, when well-structured [166]. In the same manner, a regular screening particular in vulnerable patients is also crucial to prevent the development and seriousness of MDD, as the time between the onset and help-seeking may be significantly long (even 5 years) [167]. Moreover, recent data claimed that the real efficacy of antidepressants in patients with MDD may be quite similar or attributable to those patients receiving placebo agents [168,169,170]. Conversely, the nocebo effect also represents an important challenge in the clinical management of depression, as either antidepressants or placebo agents could be associated with unexpected adverse effects [171], hence showing the need of an objective measure to evaluate the real efficacy of an antidepressant. In this sense, further approaches are being under research to facilitate the diagnosis, prognosis and prediction of MDD, including various laboratory markers, genomics, proteomics, metabolomics and multiple biological components [19,172,173]. Epigenomics as well as miRNAs are arising as a promising field of novel objective biomarkers used in MDD, although the use of these components in clinical practice are still on its infancy [174,175,176].

Firstly, among miRNAs studied with diagnostic value, there have been recorded studies about let-7, miR-16, miR-22 *, miR-24, miR-26, miR-30, miR-34, miR-101, miR-124, miR-132, miR-134, miR-135, miR-144, miR-146, miR-181a, miR-182, miR-184, miR-185, miR-193, miR-200, miR-218, miR-221, miR-451, miR-494, miR-504 and miR-1202. Secondly, miRNAs with prognostic value here reviewed are let-7, miR-9, miR-30, miR-34, miR-132, miR-137 and miR-1202. Thirdly, miRNAs with predictive value are miR-15a, miR-16, miR-24, miR-26a-2, miR-29, miR-34, miR-124, miR-133b, miR-135, miR-146a, miR-183, miR-212, miR-221, miR-451 and miR-1202. Details for each miRNA mentioned are specially detailed in Table 1. Apparently, these microRNAs biomarkers have mainly translational application and just some of them have been assessed in clinical trials.

### 4.1. miRNAs with Diagnostic Value

MiRNAs that reunite enough evidence about diagnostic value are the following. First to mention, in let-7 family, let-7b and let-7c have shown promising results in case-control studies, as they regulate 27 genes in PI3k-Akt-mTOR pathways, and being downregulated it intervenes in the route dysfunction [133]. Polymorphisms detected in promoter region of let-7 family (rs10877887 and rs13293512) determine increased MDD susceptibility [177]. miR-26a-3p is downregulated in depressed mice affecting related PTEN/PI3K/Akt pathway, implying alterations in neuronal autophagy, synaptic plasticity and survival in the dentate gyrus [134,178,179].

Considering upregulation of miR-22 is relevant in the monitoring of post-stroke patient. This miRNA might be involved in microvascular impairment associated to depression [180]. Next, elevated miR-24 serum levels, which targets oxytocin, indicates etiology of MDD [93,94]. MiR-30 family seems also interesting now that certain polymorphic variants such as miR-30e increase MDD susceptibility and others are biomarkers of late life depression (miR-30d) and PSD (miR-30a-5p) [181,182,183,184]. We find more variants of susceptibility for miR-34: rs4938723/rs28757623 polymorphisms affecting miR-34b/c genes are potential risk factors for suffering from MDD; moreover, MiR-34a is differentially expressed in MDD, bipolar disorder and schizophrenia in the anterior cingulate cortex [70,185,186,187,188]. More specifically, some variations of miR-504, rs686, affecting DRD1 regulation, have been found associated with higher depression scales [76,189].

Abnormal expression of other miRNAs as putative epigenetic signatures of MDD include downregulation of miR-16 [98,110,190], upregulation of miR-124-3p [73], miR-132, directly related to self-rating depression scale and HAM-D scale [126] and downregulated miR-135 [146]. Circulating miR-134 is in fact a good biomarker for mental disorders offering high sensitivity and specificity: MDD (79% sensitivity and 84% specificity), bipolar and schizophrenic patients (79% sensitivity and 76.5 specificity) [191]. In the same line, miR-184 is differentially expressed in MDD patients in comparison to bipolar or either bipolar or depressed patients [192]. Furthermore, depressed patients present reduced levels of blood miR-200a, miR-200b and miR-200c in comparison to healthy controls [106,193] and also reduced levels of miR-218 [116], increased levels of miR-221 [194,195,196]. MiR-451 might not be a determinant biomarker as prior works have denoted either increased or decreased serum levels of miR-451 in depressed patients [194] and miR-494 also seems controversial as it appears downregulated in depressed suicide patients but augmented in peripheral blood of depressed patients compared to controls [197,198].

For its part, circulating levels of miR-144 and miR-146a are inversely correlated with depressive symptoms and Montgomery-Åsberg Depression Rating Scale (MADRS-S) [148,199,200]. Together with all these miRNAs, miR-181a, present in stress-induced upregulation, also appears altered in MDD patients [201,202]. More case-control studies have proved statistically that miR-1202 and miR-135a have better specificity sensitivity, respectively, for diagnosis and clinical management, due to their significant decreased levels in serum from patients compared to control [203].

Finally, other potential reference markers could be miR-101 in combination with miR-93 [204]. Other preclinical models keep shedding light on miRNAome for diagnostics and etiology: for instance, dermal fibroblasts of animal models show important dysregulation of miR-185, miR-193a and miR-450 are associated to depressive phenotype [205,206].

### 4.2. miRNAs with Prognostic Value

On the one hand, let-7 family downregulated expression is inversely correlated with severity of depression and also with treatment resistance [207] MiR-9 is another biomarker of severity besides a sign of childhood maltreatment [208]. Hence both miRNAs could serve to classify MDD severity. On the other hand, upregulated miR-30 appears in post-mortem brains of patients with MDD who died by suicide. This sign underlies the 5-HT1AR palmitoylation, which seems to be related to depression-like behaviors, so it represents a point to consider for clinical strategies for the treatment [69]. Certain polymorphisms in miR-34 are categorized as more severe and related to suicide idea and cognitive dysfunction. For example, the polymorphism rs2187473 affecting miR-34c is a potential genetic risk factor for cognitive decline in MDD, as well as peripheral blood leukocytes levels of miR-34b and miR-34c were related to suicide idea [188]. MiR-137 was found downregulated by 25% in postmortem prefrontal cortex of depressed patients with suicidal behavior but further prognostic proposals have not been yet exploited [198]. Differently, upregulated expression of miR-132 seems interesting in the comorbidity of cardiovascular disease and MDD and others such as visual memory deficits, correlated with anxiety symptoms [209,210]. Similarly, miR-1202, which regulates GRM4 has been observed to have also a probable association with increased risk of suicide [78,82].

Likewise, notwithstanding this area is less established, miRNAs may be of great aid as prognostic markers in patients with MDD in relation to the risk for suffering from other diseases. For instance, prior meta-analysis and systematic reviews had found an association between MDD and Alzheimer disease (AD), suggesting the possible implication of depression as an independent risk factor of AD [211,212]. In turn, patients with advanced AD tends to have a higher prevalence of MDD [213]. Many efforts are being placed in the research o different diagnostic and prognostic markers, including miRNAs [214,215,216,217]. In this sense, Wingo et al. [218] found the relevance of miR-484 as a critical link between AD and depressive symptoms, targeting a wide variety of components involved in the synaptic transmission and regulation of synaptic plasticity. Likewise, Mendes-Silva et al. [219] denoted that 7 miRNAs (let-7d-5p, let-7f-5p, let-7g-5p, miR-26b-5p, miR-191-5p, miR-361-5p, miR-664a-3p) were common for both AD and MDD, with 45 validated target genes implicated in the proteostasis, immune-inflammatory control, genomic integrity, regulation of transcriptional activity and neurotrophic support. Finally, miR-1-3p and miR-184 appears to be differentially expressed between patients with MDD and depressed subjects that also exhibited mild cognitive impairment (MCI), an early sign of probable AD development [220]. Overall, these studies support the potential role of miRNAs as prognostic biomarkers in the onset and progression of neurodegenerative diseases such as AD.

### 4.3. miRNAs with Predictive Value

In the level of therapy response, most miRNAs have reached the preclinical phase and just some of them have been translated to clinical trials. MiR-124 targets GR, which in turn, results dysfunctional in MDD. In a rodent model, they realized that for a successful antidepressant treatment it was necessary to block miR-124 action. The treatment chosen was gypenosides, which increased GR and tyrosine receptor kinase B expression in hippocampus activating BDNF signaling but also blocking it by up-regulated miR-124 [221]. Nevertheless, miR-124 seems to have contradictory results in its modus operandi. In other mice model, stressed animals presented down-regulated levels of miR-124 in hippocampus and by restoring its expression it even inhibits depressive mechanisms such as microglial activation and lessens IL-6, IL-1β, TNF-α [145]. Hence, miR-124 alone is a parameter to consider but not a good predictive biomarker yet, although tendency in rodents is, that following a chronic unpredictable mild stress method, upregulating miR-124 worsens MDD behavior by targeting 3′ in cyclic AMP-responsive element-binding protein1 (CREB1) and BDNF in hippocampus, so it may be interesting to block it [123]. Next, miR-16 (down-regulated in MDD) is another possible predictive biomarker which targets SERT, also an important pharmacological target of SSRI antidepressants such as fluoxetine. Certain animal models have denoted that miR-16 interacts with therapeutical agents in monoaminergic neurons, being possible to maximize its expression in serotonergic raphe nuclei [222]. In addition, this miRNA modulates apoptosis and autophagy related pathways as part of the effects of SSRIs, so it entails another key point to encompass during the treatment progression [66].

Next, in animal models we find similar outcomes for miR-26a-2 and miR-29. Downregulated miR-26a-2 in a context of MDD, becomes upregulated in the dorsal raphe nucleus following antidepressant therapy whereas mir-26a KO shows poorer antidepressant response in mouse models [178]. In the same manner, downregulated miR-29b-3p in MDD and overexpression in prefrontal cortex after treatment acts as a critical mediator of ketamine’s antidepressant effect in depressive rats [79]. Conversely, physical electroconvulsive stimulation in rat studies has shown altered miR-212 levels either in the blood or dentate gyrus both after acute and chronic administration, associated to BDNF regulation [223,224]. In contrast, miR-135, which has anti-inflammatory actions in brain, targeting multiple inflammatory mediators in hippocampus (IL-1β, IL-6 TNF-α and TLR-4) is critical for serotonin regulation in raphe nuclei. In presence of antidepressant drug, serum levels rise and synergistically offer antidepressant effects, being this an optimal response to the drug [71,72,146,203].

Some miRNAs proposed as predictive biomarkers have also been evaluated in MDD patients. MiR-15a shows significant up-regulation at 3–6 h post-treatment with dexamethasone (GR agonist) in peripheral blood cells of young healthy men. Due to the implication of miR-15a in the activation of stress system and resilience to stress, this miRNA as biomarker has predictive value [225]. For its part, miR-1202 has allowed to make treatment decisions. In cohort studies, responders to antidepressant treatment had lower baseline miR-1202 levels compared to non-responders [226]. However, robust blood markers of antidepressants response are miR-24-3p and miR-146a-5p, MIR-146b-5p and miR-425-3p crucial regulators of MAPK/Wnt signaling pathways which are dysregulated in MDD and significantly changed 8 weeks after treatment with duloxetine [136,227]. MiR-133b in hippocampus has shown the same outcome in the case of fluoxetine treatment but in mice [122,228].

A regulator of BDNF, miR-183 has been investigated in case-control studies before and after the intake of SSRIs, resulting increased after four weeks of antidepressant treatment [229]. Finally, Responder patients show decreased serum miR-34a and miR-221-3p, which were directly correlated with a decrease in HAM-D score [187]. A positive relationship between paroxetine effectiveness and miR-451 was found in patients [187] although the effect of ketamine in miR-451 showed in rats and patients still controversial results [83,230].

## 5. miRNAs as Promising Therapeutic Targets of MDD

In this case, miRNAs-based therapy has been receiving great attention during the last years in a broad spectrum of diseases including MDD [231,232,233]. However, most of the studies are preclinical and to our knowledge there are no clinical trials conducted in the therapeutic use of MDD, and many efforts are needed to progress in this field. Strategies to use miRNAs as therapeutic targets consist of the upregulation or downregulation of a particular miRNAs of interest, using oligonucleotides to mimic or inhibit miRNA expression or small molecules to increase or suppress miRNA function [234] These molecules need to be transfected by using viral vectors encoding miRNA mimics/antagonists, nanoparticles such as liposomes, or cell-derived vesicles (exosomes, microvesicles, retroviral and apoptotic bodies) [22,235]. In vivo studies require an efficient delivery of the miRNAs, which may avoid degradation by RNAse, improve targeting accuracy and prevent an undesired immune rejection. Accordingly, intravenous injection or local treatment were the main methods of administration for in vivo miRNA delivery, although there are some studies evaluating the possible use of oral and intranasal administration [236,237]. In the background of MDD miRNAs that may interact with therapeutic agents or be potential targets are let-7, miR-9, miR-16, miR-18, miR-26a-2, miR-30, miR-34, miR-96, miR-101, miR-124, miR-128, miR-132, miR-133b, miR-134, miR-135, miR-144, miR-146a, miR-155, miR-182, miR-221, miR-323 and miR-451. Compelling details for each miRNA mentioned are collected in Table 1.

Amelioration of dysregulated let-7 family is being studied from the epigenetic intervention view by physical activity [238]. Other possible translational applications are inspired by lentiviral-mediated let-7d overexpression with anxiolytic and antidepressant effects [75,105,207] Following the same technology, lentiviral injection of miR-144 exerted antidepressant roles in the hippocampus of chronic unpredictable mild stress rats [121].

Animal models keep defying targets towards the reversion of depressive-like behaviors. Notch 1 activation for neural connectivity and synaptic plasticity is a desire in which miR-9 is wanted to be blocked [239,240,241]. In the same line, blocking miR-18a would be suitable to restore downregulation of GRs [118]. Moreover, miR-132 inhibition increased BNDF levels in in vivo results [242,243] and also blocking miR-134 in vivo ameliorated neuronal structural abnormalities, biochemical changes and depression-like behaviors. Inhibition of miR-134 together with the use of a GR antagonist and SIRT-1 agonist depression susceptibility induced by prenatal dexamethasone exposure (PDE) in offspring rats could be prevented [90]. MiR-182 inhibition in the hippocampus also seems to exert antidepressant effects in vivo [244]. In vivo KO and upregulation of miR-323 was associated with increased anxiety and augmented emotionality [245].

For its part, antagomiR-34a activated TrkB/MEK1/ERK signaling and improved spine morphology in the hippocampus, exerting antidepressant effects [187] Targets in ERK pathway also seem to regulate miR-221 expression, which was positively associated with MDD pathogenesis [246].

The use of miR-96 antagonists led to a reduced pro-inflammatory and pathogenic marker related with MDD [141]. In vivo inhibition of miR-124 conducted in different animal models and brain regions show some promising results. In the panorama of in vitro studies, potential posttranscriptional switching mechanism in the amygdala targeting miR-128 may benefit Wnt restoring [138].

In contrast, there are some miRNAs with antidepressant effects according to several animal models: miR-26a-2 targets HTR1A in serotonergic neurons, easing stress resilience [134,178,179]. Following, miR-101 mimics reversed depressive-like behaviors in CUMS rats [120]. Similarly, miR-146a mimic treatment inhibited microglial activation besides TNF-α, IL-1β, IRAK1 and TRAF6 expression in BV-2 cells [149].

MiR-133b augmentation was associated with decreased apoptosis, repressed inflammatory reaction, and increased expression of GFAP, BDNF and neurotransmitters in hippocampal tissues of depression rats [122]. Similarly, growing evidence from animal models have also reported the adjuvant antidepressant action in combination with antidepressant drugs. Combined use of miR-135a mimic plus antidepressants (fluoxetine) induced a significant decrease of pro-inflammatory markers in CUMS mice [72].

Above mentioned miR-144, which contributes to antidepressive-like mood is lower in MDD patients. In order to increase its expression, animal models have validated mood stabilizers such as lithium and sodium valproate [247]. Conversely, there have been some miRNAs that interfere with antidepressant treatments. The case of miR-16 is an antagonist [222]. Other treatments have simply been useful to observe and prevent long-term effects in the expression of stress-related miRNAs such as miR-30a with lurasidone [183].

## 6. Future Directions: miRNAs Modulation through Lifestyle Interventions

Although all these mentioned potential therapies have shown promising results and elucidated the mechanisms of certain miRNAs in the pathogenesis and prognosis of MDD, we ought to consider that, in clinical practice, it is possible to apply certain strategies based on lifestyle factors that effectively have demonstrated to regulate inflammation, immune system function, metabolism and mood. Nowadays, thanks to the rising evidence about epigenetics regulation and good habits, research for MDD is focusing also on physical exercise, dietary interventions and mindfulness as fields of growing interest. Physical exercise is considered a perfect anxiolytic for mental disorders [248]. Preclinical studies and then clinical studies corroborate that physical therapy is a perfect non-pharmacological adjuvant. CUMS depressed mice subdued to aerobic exercise have shown increased hippocampus expression of miR-223 and inhibition the TLR4/MyD88-NFκB signaling pathway and associated-inflammatory response, IL-1β levels, and increasing IL-10 levels [249]. These neuroimmunomodulatory effects have been confirmed in MDD patients: the downregulation of proinflammatory cytokines, M1 microglia and reactive astrocytes [250,251].

Studies in MDD patients declare that not only systemic association can be attenuated but also emotional behavior can be modulated by physical exercise. Hippocampal neuroplasticity can be positively regulated by exercise, promoting the downregulation or upregulation of many of these miRNAs [252]. Hence exercise can be considered as a neuroprotector now that it can repress the expression of certain microRNAs related to damage in brain [253,254] and age-related hippocampal deterioration and depressive symptoms. At the same time, other biomolecules that are dysregulated in MDD are restored with exercise. For example, the increase of orexin-A allows hippocampal homeostasis in neurogenesis [255]. It is also a strong instigator of BDNF expression and recent studies allege that it can synergize with antidepressant treatment enhancing their actions [256].

As many authors consider, MDD is a mind-body disorder, therefore, combining physical activity with mindfulness techniques are on the road of holistic approaches; in this sense, yoga seems to have the key combination [257]. In this context, relaxation strategies every time are offering more beneficial outcomes in clinical trials. Levels of macrophage migration and related regulating miRNAs, such as miR-451a, significantly change from baseline to psychotherapeutic interventions that include meditation, although psychiatric symptoms have not been widely associated and further research is still required [258]. These results seem to function in different groups of age, even in elderly, where recent mindfulness approaches have expounded that it can prevent cognitive impairment and neuronal loss by increasing neuronal expression of miR-29c, downregulating STAT3 in hippocampus [259].

Finally, and not less important, dietary interventions are of note in all this panorama. Epigenetic modulation and metabolic reprogramming by diet and specifically, treating nutritional deficiencies with nutraceuticals supplementation or specific interventions with certain functional foods denote maximized benefits in patients with MDD [13]. In vitro and in vivo studies have collected data about dysregulation of miRNAs and the impact of diet on this situation. Some examples found in psoriasis studies are the downregulation of miR-155 by flavonoids such as quercetin and by vitamin D; or the upregulation of miR-125b by vitamin D and selenium [260]. However, the knowledge about diet-miRNAs in MDD is still limited, although its pathophysiology shares common malnutrition with other inflammatory diseases, mainly based on the lack of micronutrients minerals and vitamins, which play decisive roles in immunomodulation and therefore immunonutrition. The use of probiotics and prebiotics either in the food or through commercial formulas (i.e., Ecologic^®^ barrier) may be an interesting approach to regulate host miRNA expression, gut microbiota and the inflammatory status of patients with established diseases [261].

## 7. Conclusions

miRNAs are critical epigenetic modulators of a wide variety of functions in the brain, also playing a key role in MDD pathogenesis. Throughout the whole manuscript the relevance of miRNAs has been reviewed as promising diagnostic, prognostic and predictive markers, also collecting preclinical studies evaluating the potential benefits from using miRNAs as treatment targets. Moreover, lifestyle interventions are central regulators of miRNA expression, opening diverse therapeutic windows that may be of great aid in the field of MDD. However, much more efforts are needed in all these fields, especially in the clinical translation and applications of miRNAs in MDD, as they may be the present and future of personalized medicine in those patients.

**Table 1:** A summary of the different miRNAs up or downregulated involved in the pathophysiology of depression, as well as their potential translational applications.

miRNA	Upregulated/ Downregulated	Mechanism Involved/Effects	Translational Applications	References
1. Let7	Downregulated	Let-7d targets D3R in the hippocampus Let7b and let7c regulates PI3k-Akt-mTOR pathway IL-6 lead to a dysregulation of Let-7 family in depressive rat models	Diagnostic: rs10877887 and rs13293512 polymorphisms affecting Let-7 family are associated with increased MDD susceptibility Prognostic biomarker: Lower expression of Let-7 family correlates with higher degree of severity and treatment resistance Therapeutic: Let-7 family dysregulation could be ameliorated by epigenetic interventions such as physical activity Lentiviral-mediated Let-7d overexpression is associated with anxiolytic and anti-depressant-like action	[75,105,133,177,207,238]
2. miR-9	Upregulated	Increased levels of miR-9 affect intrinsic amygdala functional connectivity, related to depressive severity and childhood maltreatment	Prognostic: To classify MDD severity Therapeutic: Animal models show: an inhibitor may block neuronal apoptosis activating Notch 1 signaling pathway, which is key for neural development and brain homeostasis (neuronal connectivity, synaptic plasticity and learning/memory). Notch signaling pathway is crucial in early neurodevelopment and late-life neurodegeneration.	[208,239,240,241,262]
3. miR-15a	Downregulated in the amygdala	Post-transcriptional regulation of SERT with miR-16. Reduced levels of amygdalar miR-15 but elevated circulating levels in peripheral blood increase anxiety-like behaviors.	Predictive: significant up-regulation at 3–6 h post-treatment with dexamethasone	[65,111,225]
4. miR-16	Upregulated	Post-transcriptional regulation of SERT with miR-15a Depression induced by maternal deprivation but not CUPS was significantly associated with miR-16 upregulation, probably targeting BDNF in the hippocampus MiR-16 regulate apoptosis and autophagy and could account for some part of the therapeutic effect of SSRIs	Diagnostic: CSF levels of miR-16 is downregulated in patients with MDD Predictive: By reducing miR-16, serotonergic functions in noradrenergic neurons improve when combined with antidepressants Therapeutic: Although inconsistencies within different animal models cannot conclude if miR-16 is involved in affective disorders, it is clear that it interferes with antidepressant treatments, antagonizing them.	[65,66,98,110,190,222]
5. miR-18a	Downregulated	Implied in resilience to stress, miR-18a-5p acts as a negative epigenetic regulator of 5-HT1AR, altering the serotonergic homeostatic balance and functioning of the hippocampus. The upregulation of miR-18a not only in hippocampus, but also in prefrontal cortex, is related to downregulation of GRs and depressive-like behaviors	Therapeutic: Possible new antidepressant strategies aiming to restore this biological processes.	[118,119]
6. miR-22	Upregulated	miR-22 might be involved in cerebral microvascular impairment associated to depression	Diagnostic: miR-22 levels are potential indicators of post-stroke depression	[180]
7. miR-24	Upregulated	MiR-24 targets oxytocin, which may be related to MDD etiopathogenesis MiR-24-3p is associated with the regulation of Wnt and MAPK signaling pathways,	Diagnostic: Elevated miR-24 serum levels is an indicator of MDD Predictive: Serum levels of miR-24 is a robust blood marker of antidepressants response	[93,94,136,263]
8. miR-26	Downregulated	MiR-26a-3p is a critical regulator of PTEN/PI3K/Akt pathway, regulating neuronal autophagy, synaptic plasticity, and survival in the dentate gyrus of a rat model of depression	Diagnostic: serum miR-26 is altered in depressed mice Predictive: miR-26a-2 levels are significantly upregulated in the dorsal raphe nucleus following antidepressant therapy and mir-26a KO shows poorer antidepressant response. Therapeutic: miR-26a-2 functions as an endogenous antidepressant by targeting HTR1A in serotonergic neurons.	[134,178,179]
9. miR-29	Downregulated	MiR-29 is essential for neuronal survival in the brain, targeting VDAC1 MiR-29 is highly produced by astrocytes and their dysregulation in MDD may be an indicator of abnormal functioning of these cellsMiR-29b-3p is critical for inhibiting GRM4 expression in the prefrontal cortex of depressive-like rats MiR-29 is a major regulator of endoplasmic reticulum stress in neurons, a crucial pathophysiological event in the neurons related to MDD	Predictive: Overexpression of miR-29 acts as a critical mediator of ketamine’s antidepressant effect in depressive rats	[55,79,101,102,264]
10. miR-30	Upregulated	miR-30 family miRNAs mediate chronic stress-induced depression-like phenotype by altering hippocampal neurogenesis, neuroplasticity, epigenetic and transcriptional regulators such as Mll3 and Runx1 Socs3, Ppp3r1, Gpr125, and Nrp1. MiR-30a regulates neural nutrient signaling pathway, axon guidance, insulin and other signaling pathways The polymorphism miR-30e ss178077483 is associated with P300 latency and the individuals with the C/T genotype have a longer P300 latency than those carrying the C/C genotype	Diagnostic: polymorphic miR-30e variant ss178077483 appears to increase MDD susceptibility miR-30a-5p is a diagnostic marker of PSD miR-30d is associated with late life depression Prognostic: Elevated levels of miR-30e was detected in post-mortem brain samples of patients with MDD died by suicide Therapeutic: The use of lurasidone during adolescence was able to prevent the up-regulation of miR-30a and normalized the expression of its target genes in response to prenatal stress exposure.	[69,113,181,182,183,184]
11. miR-34	Upregulated	LPS and stress, both related to MDD induce the expression of miR-34a MiR-34a targets synaptotagmin-1 and Bcl-2 associated with neuronal spine damage MiR-34a induce depressive-like behavior and impact in the serotoninergic activity in the raphe nuclei of mice Patients with MDD show a significant decrease in NOTCH signaling components, inversely related to miR-34b/c expression	Diagnostic: rs4938723/rs28757623 polymorphisms affecting miR-34b/c genes are potential risk factors for suffering from MDD MiR-34a is differentially expressed in MDD, bipolar disorder and schizophrenia in the anterior cingulate cortex. Prognostic: There is a significant association between rs4938723 and negative life events in relation to MDD risk. The polymorphism rs2187473 affecting miR-34c is a potential genetic risk factor for cognitive decline in MDD Peripheral blood leukocytes levels of miR-34b and miR-34c were related to suicide idea and cognitive function Therapeutic: antagomiR-34a activated TrkB/MEK1/ERK signaling and improved spine morphology in the hippocampus., exerting antidepressant effects Predictive: Responder patients show decreased serum miR-34a-5p, a miRNA directly correlated with HAM-D score	[64,168,170,171,214,265]
12. miR-96	Upregulated	miR-96 targets SV2C in the CA1 area of the hippocampus, leading to a depressive-like behavior and memory impairment	Therapeutic The use of miR-96 antagonists led to a reduced pro-inflammatory and pathogenic markers related with MDD	[141]
13. miR-101	Downregulated	CUMS rats present reduced miR-101 in the ventrolateral orbital cortex with increased dual specificity phosphatase 1 (DUSP1) expression and reduced ERK-1 and BDNF Flinders sensitive line rats presented decreased miR-101b expression in the prefrontal cortex targeting the neuronal glutamate transporter SLC1A1.	Diagnostic: miR-101 in combination with miR-93 are potential reference biomarkers of MDD Therapeutic: miR-101 mimics reversed depressive-like behaviors in CUMS rats	[80,120,204]
14. miR-124	Upregulated	miR-124-3p is a master regulator of multiple processes targeting SOX-9, JAG-Notch, CREB-1, SIRT-1 BDNF, Ezh2, SCP1, GR and neuroinflammatory components.	Diagnosis: miR-124-3p is upregulated in patients with MDD Predictive: miR-124 reduced levels in plasma could be an indicator of favorable antidepressant response Therapeutic: In vivo inhibition of miR-124 conducted in different animal models and brain regions show some promising results	[81,89,103,104,123,145,266,267]
15. miR-128 family	upregulated	MiR-128-3p is involved in Wnt downregulation in the amygdala of learned helpless rats and patients with MDD Patients with type 2 diabetes mellitus and depression show increased levels of miR-128, cortisol while reduced BDNF and shortened telomeres	Therapy: Potential posttranscriptional switching mechanism in the amygdala targeting miR-128 may benefit Wnt restoring	[125,138]
16. miR-132	Upregulated	MeCP2, regulation of BDNF hippocampal levels Higher miR-132 levels are associated with both lower fractional amplitude of low frequency fluctuations and lower grey matter volume in fronto-limbic network; as well as poorer cognitive performance in attention and executive function Currently considered a neurimiRs	Diagnosis: There is a direct relationship between miR-132 expression levels and self-rating depression scale and HAM-D scale Prognostic: miR-132 may play a role in the coexistence of cardiovascular disease and MDD. Increased miR-132 expression levels were associated with visual memory deficits, correlated with anxiety symptoms Therapeutic: miR-132 inhibition increased BNDF levels in vivo	[126,209,210,242,243,266,268]
17. miR-133b	Downregulated	CGTF suppression	Predictive: miR-133b increase in the hippocampus of mice after fluoxetine treatment Therapeutic: miR-133b augmentation was associated with decreased apoptosis, repressed inflammatory reaction, and increased expression of GFAP, BDNF and neurotransmitters in hippocampal tissues of depression rats	[122,228]
18. miR-134	Upregulated,	UCMS exposure significantly increased the expression of miR-134 within the ventromedial prefrontal cortex, leading to a decrease in Limk1 and cofilin.	Diagnostic: miR-134 is downregulated in patients with MDD in comparison to healthy controls (79% sensitivity and 84% specificity), bipolar and schizophrenic patients (79% sensitivity and 76.5 specificity). Therapeutic: Blocking miR-134 in vivo ameliorated neuronal structural abnormalities, biochemical changes and depression-like behaviors. Inhibition of miR-134-5p together with the use of a GR antagonist and SIRT-1 agonist depression susceptibility induced by prenatal dexamethasone exposure (PDE) in offspring rats could be prevented	[90,114,191]
19. miR-135	Downregulated	MiR-135 exert anti-inflammatory actions in the brain targeting multiple inflammatory mediators in the hippocampus (IL-1β, IL-6 TNF-α and TLR-4) MiR-135 is critical for serotonin regulation in the raphe nuclei	Diagnostic: Downregulated in patients with MDD Predictive: circulating miR-135 levels could be an indicator of favorable response to antidepressants. Therapeutic: Combined use of miR-135a mimic plus antidepressants (fluoxetine) induced a significant decrease of pro-inflammatory markers in CUMS mice	[71,72,146,203,269]
20. miR-137	Probably downregulated	miR-137 levels are significantly diminished in the brain in post-stroke depression rats, miR-137 is related to anxiety behaviors in mice	Prognostic: miR-137 levels are substantially down-regulated by 25% in the postmortem prefrontal cortex of depressed patients with suicidal behavior Potential diagnostic, therapeutic and prognostic factor still unexplored	[198,270,271]
21. miR-138	Upregulated	MiR-138 regulate depression-like behavior by targeting by SIRT1 expression in the hippocampus.	Not studied yet	[91]
22. miR-144	Downregulated	Low MiR-144-5p is associated with multiple neuroinflammatory proteins MiR-144 targets PTP1B activating the TrkB/BDNF signaling in the hippocampus	Diagnostic: Circulating miR-144-5p levels are inversely correlated with depressive symptoms and Montgomery-Åsberg Depression Rating Scale (MADRS-S) Therapeutic: Lentiviral injection of miR-144 exerted antidepressant roles in the hippocampus of chronic unpredictable mild stress rats. Mood stabilizers (lithium and sodium valproate) increases miR-144 levels in animal models.	[121,148,199,247]
23. miR-146	Downregulated	MiR-146a reduces neuroinflammation and depressive behavior in mice models through targeting Iba-1, iNOS, IL-1β, TNF-α, interleukin 1 receptor associated kinase 1 (IRAK1), TNF receptor-associated factor 6 (TRAF6) and phosphorylated NF-κB p65 MiR-146 is related to Wnt signaling, Cancer, Endocytosis, Axon guidance and MAPK signaling	Diagnostic: miR-146a expression before treatment is inversely correlated with HAM-D score Therapeutic: miR-146a mimic treatment inhibited TNF-α, IL-1β, IRAK1 and TRAF6 expression in BV-2 cells Predictive: miR-146a may be a promising marker to predict fluoroxetine and antidepressant response in patients with MDD.	[136,149,200,207,227]
24. miR-155	Upregulated	miR-155 inhibits MyD88 gene alleviating depressive-like behaviors miR-155 is involved in neuroinflammatory pathways (IL-6 and TNF-α raise) and ciliary neurotrophic factor expression. MiR-155 is associated with increased apoptosis in the hippocampus by targeting Wnt/β-catenin signalling and microglia	Therapeutic: miR-155 deletion reduces anxiety and depressive-like behavior in vivo; Citalopram and other antidepressants appears to downregulate miR-155 expression miR-155 has been proposed as an interesting approach in treatment-resistant depression. Diagnostic: Cellular and CSF levels of miR-155 are upregulated in patients with MDD However there is still controversy regarding serum levels of miR-155 miR-155 before treatment was directly correlated with severity of depression.	[92,127,137,142,143,207,272]
25. miR-181	Probably upregulated	MiR-181a appears to be associated with stress-induced upregulation of the noradrenergic phenotype, observed in depressive patients, also regulating GRs; PI3K/Akt/mTOR pathway	Diagnostic: miR-181a together with 32 additional miRNAs were altered in patients with MDD in comparison to controls.	[201,202]
26. miR-182	Upregulated	Abnormal processing of miR-182 in the dentate gyrus of the hippocampus in individuals carrying appears to contribute to the dysregulation of circadian rhythms in MDD patients with insomnia targeting CLOCK gene miR-182 is inversely correlated with BDNF levels	Diagnostic: T allele of the rs76481776 polymorphism may be related to clinic insomnia; miR-182 is inversely correlated with self-rating depression score Therapeutic: miR-182 inhibition in the hippocampus may exert antidepressant effects in vivo.	[84,126,244,273]
27. miR-183	Upregulated	Inhibition of BDNF activity in the brain. MiR-183 is upregulated in the hippocampus of chronic unpredictable mild stressed rats.	Predictive: miR-183 is increased after 4 weeks of antidepressant (escitalopram) therapy	[115,229]
28. miR-184	Downregulated	MiR-184 targets PDE4B NCOR2 in the anterior cingulate cortex of depressed patients KO models of flies demonstrate that reduced levels of miR-184 is associated with a depressive behavior, more prominent in older flies	Diagnostic: MiR-184 is differentially expressed in MDD patients in comparison to bipolar or either bipolar and depressed patients	[192,274]
29. miR-200	Upregulated	Upregulated in the hippocampus of rodents affected by maternal separation and inescapable shock targeting Zeb1 and Zeb2 MiR-200a appears to be involved in altered neurogenesis, inflammatory activation, lipid metabolism, disturbed circadian rhythm, and insulin secretion in the co-existence of pain and depression	Diagnostic: Depressed patients present reduced levels of blood miR-200a, miR-200b and miR-200c in comparison to healthy controls.	[106,193,275]
30. miR-212	Upregulated	Regulation of a plethora of neuronal signaling pathways and morphogenesis Both acute and chronic stress exposure lead to miR-212 augmentation in the hippocampus and the amygdala, related to anxiety behaviors	Predictive: Electroconvulsive stimulation raises miR-212 levels either in the blood or dentate gyrus both after acute and chronic administration MiR-212 is also proposed as a promising predictor of serotonin reuptake inhibitors	[220,223,224,243]
31. miR-218	Downregulated	miR-218 acts as a molecular switch that may determine susceptibility vs. resilience to chronic stress acting through corticosterone-related networks	Diagnostic: Plasma levels of miR-218 appears to be diminished in patients with MDD	[116,196,276]
32. miR-221	Upregulated	MiR-221 is positively associated with MDD pathogenesis in rats with chronic unpredictable mild stress by targeting Wnt2/CREB/BDNF pathway	Diagnostic: Serum and cerebrospinal fluid of depressed patients show increased levels of miR-221; elevated serum levels of miR-221-3p can be used as an indicator for depressed mood in perioperative patients; Increased serum and CSF levels is a major risk factor of PSD, directly correlated with HAM-D score, IL-6 and TNF-α levels. Predictive: Paroxetine may lead to decreased serum levels of miR-221-3p, which is related to decreased HAM-D score Therapy: ERK pathway regulates miR-221 expression	[124,187,194,195,246,277]
33. miR-323	Downregulated	MiR-323 appears to be diminished in depression animal models, but upregulated in the brain of newborns after prenatal stress Targets and affected regions: Erb-b2 receptor tyrosine kinase 4 (ERBB4), neuregulin pathway, hippocampus, anterior cingulate cortex and habenula	Therapeutic: In vivo knock-down and upregulation of miR-323 was associated with increased anxiety and augmented emotionality.	[245,278,279]
34. miR-376	Upregulated	A shift in the expression of miR-376c-3p, miR-455-3p and miR-337-3p in the synaptic fraction over total fraction was found in MDD subjects compared with healthy controls. MiR-376b-5p probably targets a number of genes relevant to stress signaling pathways and neuronal regulation, being downregulated after augmented maternal care (Kcnh5 ↓, Sh3rf2↓, Acox2↓, Otx2↓, Myof↓, Frrs1↓, Dio2↓, Acss3) miR-376b and miR-208 increased whereas miR-9-1 decreased under acute and chronic stress conditions,	Not studied yet.	[97,105,117,280]
35. miR-429	Upregulated	miR-429 appears to be upregulated after acute and repetitive stress exposure miR-429 is upregulated in the fibroblasts of depressed patients, affecting to the expression of 842 genes in which 9 are known to be involved in MDD pathogenesis.	Not studied yet.	[85,281,282,283]
36. miR-451	Downregulated	Maternal deprivation induced downregulation of miR-451 in the hippocampus. MiR-451 appears to regulate some critical genes associated to MDD including CREB pathway, GABAergic and cholinergic neurotransmission	Diagnostic: Prior works have denoted either increased or decreased serum levels of miR-451 in depressed patients Prognostic: There is an inverse relationship between miR-451 levels and HAMD score Predictive: A positive relationship between paroxetine and fluoexetine effectiveness and miR-451 was found, although the effect of ketamine in miR-451 is still controversial	[83,133,187,194,230]
37. miR-494	Downregulated	miR-494 is downregulated in the dorsolateral prefrontal cortex depressed suicide patients	Diagnostic: miR-494 is augmented in the peripheral blood of depressed patients in comparison to healthy controls.	[197,198,276]
38. miR-504	Upregulated	miR-504 inhibits dopamine D1 and D2 receptor gene (DRD1/2) miR-504 is increased in the nucleus accumbens of animals with maternal deprivation, leading to chronic unpredictable stress and higher sensitivity to stress in the adulthood	Diagnostic: rs686 polymorphism affecting DRD1 miR-504 regulation is associated with higher depression scales	[76,77,189]
39. miR-874	Upregulated	MiR-874-3p influence IDO1 activity and its effect on lipopolysaccharide (LPS)-induced depression-like behavior in mice	Not studied yet.	[155]
40. miR-1202	Downregulation	Regulation of glutamatergic, dopaminergic, GABAergic, and serotonergic neurotransmission	Diagnosis: miR-1202 is reduced in patients with MDD Predictive: miR-1202 predicts citalopram treatment response Responder patients displayed lower baseline miR-1202 levels than nonresponders. Prognosis: Probable association with increased risk of suicide.	[78,82,203,226]

## Data Availability

Not applicable.
